# Strategies for the recovery of active proteins through refolding of bacterial inclusion body proteins

**DOI:** 10.1186/1475-2859-3-11

**Published:** 2004-09-02

**Authors:** Luis Felipe Vallejo, Ursula Rinas

**Affiliations:** 1 Biochemical Engineering Division, GBF German Research Center for Biotechnology, Mascheroder Weg 1, 38124 Braunschweig, Germany

## Abstract

Recent advances in generating active proteins through refolding of bacterial inclusion body proteins are summarized in conjunction with a short overview on inclusion body isolation and solubilization procedures. In particular, the pros and cons of well-established robust refolding techniques such as direct dilution as well as less common ones such as diafiltration or chromatographic processes including size exclusion chromatography, matrix- or affinity-based techniques and hydrophobic interaction chromatography are discussed. Moreover, the effect of physical variables (temperature and pressure) as well as the presence of buffer additives on the refolding process is elucidated. In particular, the impact of protein stabilizing or destabilizing low- and high-molecular weight additives as well as micellar and liposomal systems on protein refolding is illustrated. Also, techniques mimicking the principles encountered during *in vivo *folding such as processes based on natural and artificial chaperones and propeptide-assisted protein refolding are presented. Moreover, the special requirements for the generation of disulfide bonded proteins and the specific problems and solutions, which arise during process integration are discussed. Finally, the different strategies are examined regarding their applicability for large-scale production processes or high-throughput screening procedures.

## Background

Recombinant DNA technology made available several simple techniques for transferring and efficiently expressing desired genes in a foreign cell. Thus, it was thought that unlimited and inexpensive sources of otherwise rare proteins would become accessible. It soon was observed that the host cell had a great influence on the quality and quantity of the produced recombinant protein. For example, recombinant protein production in mammalian cells yields a biologically active protein with all the required posttranslational modifications. However, mammalian cell cultivation is characterized by low volumetric yields of the recombinant protein, long cultivation times and requirements for expensive bioreactors and medium components. All these points have a great impact on the production costs. On the other hand, bacterial cultivation processes are based on inexpensive media in which fast growth and high cell concentrations can be obtained. These high cell concentrations combined with higher production rates of the bacterial expression system result in higher volumetric productivities. However, the production of recombinant proteins in bacteria such as *Escherichia coli *frequently yields an inactive protein, aggregated in the form of so-called inclusion bodies.

Though, producing an inactive target protein in the form of inclusion bodies is an important drawback, it also has several advantages such as the high degree of purity of the target protein in the aggregate fraction and the increased protection from proteolytic degradation compared to the soluble counterpart. Inclusion bodies have long been considered completely inert towards *in vivo *dissolution; only recently it was shown that proteins can be resolubilized *in vivo *from inclusion body deposits [1]. Although inclusion bodies in general consist of inactive proteins, *E. coli *can be the superior expression system compared to eukaryotic expression systems when the activity of the recombinant protein can be regained through refolding from the produced inclusion bodies. However, one needs to consider that the decision to select a specific expression system frequently is based on more trivial reasons such as staff knowledge and available equipment and facilities of the producing company/institute.

A good example to demonstrate the diverse routes that can be used for recombinant protein production is the manufacturing of tissue-type plasminogen activator (tPA). This protein enables the dissolution of blood clots and is used therapeutically for the treatment of myocardial infarction, thrombosis, pulmonary embolism, and strokes. To assure sufficient tPA for such a widespread application, an economic production process is a necessity. From the beginning, both the mammalian as well as the microbial route were explored for the production of tPA [2]. tPA is a fairly large (527 amino acids) monomeric protein containing 17 disulfide bridges. Because of this complexity, tPA was first produced in *E. coli *in the form of inclusion bodies while the mammalian expression system yielded an active protein that was secreted into the culture medium. More recently, obtaining active tPA through secretion into the periplasm of *E. coli *was attempted [3-5]. The early unsatisfactory yields have been improved [6,7] rendering the *E. coli *secretion system as a future potential alternative route to generate functional tPA. Other recombinant organisms such as yeast [8], fungi [9] or insect cells [10] have not yet been considered as industrial producers for this protein.

Initially, the recombinant tPA introduced into the market was obtained from genetically engineered mammalian cells [2]. At that time, generating biologically active tPA from *E. coli *produced material was a process with a poor overall yield [2]. Today, the majority of commercial tPA (alteplase, Activase^®^) is still produced using a mammalian expression system (Genentech: ). In addition, an amino substituted tPA produced by the mammalian expression system with increased half-life (tenecteplase) was developed. Alternatively, a non-glycosylated, truncated tPA (reteplase, Retavase^®^) produced in *E. coli *in form of inclusion bodies and afterwards refolded to its biologically active form is now on the market (Centocor: ) and apparently gains market share at the cost of the mammalian-derived product(s) (see Genentech 2004 First Quarter Report).

Thus, continuous research effort focused on developing new refolding techniques or improving existing ones by including novel refolding aiding agents can make the bacterial inclusion body system an excellent alternative to the mammalian expression system or other expression systems that can directly generate active proteins with a complex disulfide bond structure. The foremost aim in improving protein refolding from *E. coli *produced inclusion bodies is to increase both the allowed protein concentrations during the refolding process and the final refolding yield. Recent advances in this area are summarized in conjunction with a short overview on inclusion body isolation and solubilization procedures. Moreover, the different techniques are discussed regarding their applicability for large-scale production processes or high-throughput screening procedures.

### Isolation and solubilization of inclusion bodies

A high degree of purification of the recombinant protein can be achieved by inclusion body isolation [for recent reviews on various aspects of inclusion body formation and renaturation of inclusion body proteins please refer also to [11-18]]. Inclusion bodies are in general recovered by low speed centrifugation of bacterial cells mechanically disrupted either by using ultrasonication for small, French press for medium, or high pressure homogenization for large scale. Main protein contaminants in the crude inclusion body fraction are proteins from the cell envelope, the outer membrane proteins [19]. These proteins are not integral inclusion body contaminants but coprecipitate together with other insoluble cell material during inclusion body recovery. Lysozyme-EDTA treatment before cell homogenization facilitates cell disruption. Addition of detergents such as Triton X-100 and/or low concentrations of chaotropic compounds either prior to mechanical cell breakage or for washing crude inclusion body preparations allow the removal of membrane proteins or other nonspecifically adsorbed cell material [11-14].

After their isolation, inclusion bodies are commonly solubilized by high concentrations of chaotropic agents such as guanidinium hydrochloride or urea. Although expensive, guanidinium hydrochloride is in general preferred due to its superior chaotropic properties. Moreover, urea solutions may contain and spontaneously produce cyanate [20], which can carbamylate the amino groups of the protein [21]. In addition, inclusion body solubilization by urea is pH dependent and optimum pH conditions must be determined for each protein [22]. There are also reports that inclusion bodies can be solubilized at extreme pH in the presence or absence of low concentrations of denaturants [23-25]. However, extreme pH treatments can result in irreversible protein modifications such as deamidation and alkaline desulfuration of cysteine residues [26]. Finally, inclusion bodies can be solubilized with different types of detergents [27,28], low concentrations of denaturants [29,30], or even by utilization of the aggregation suppressor arginine [29]. Inclusion body proteins solubilized under these mild conditions can possess a native-like secondary structure [28-30], and may even reveal some biological activity [29,31]. It has also been demonstrated that the utilization of milder solubilization conditions can lead to higher final refolding yields compared to solubilization by high concentrations of guanidinium hydrochloride or urea [27].

In addition to the solubilizing agent, the presence of low molecular weight thiol reagents such as dithiothreitol (DTT) or 2-mercaptoethanol is generally required. These substances will reduce nonnative inter- and intramolecular disulfide bonds possibly formed by air oxidation during cell disruption and will also keep the cysteines in their reduced state [14,15]. Optimum conditions for disruption of existing disulfide bonds are found at mild alkaline pH since the nucleophilic attack on the disulfide bond is carried out by the thiolate anion. Residual concentrations of reducing substances can negatively affect the refolding process, thus, they are frequently removed (*e.g*. by dialysis) before starting the refolding procedure. As an alternative, immobilized reducing agents (*e.g*. DTT; VectraPrime™, Biovectra) could simplify reducing agent removal by centrifugation after the solubilization process. Finally, the pH must be reduced before the removal of the reducing agent from the solution containing the solubilized protein to prevent the formation of undesired disulfide bonds.

### Principles of refolding solubilized and unfolded proteins

#### Correct refolding versus aggregation

**Figure 1:**
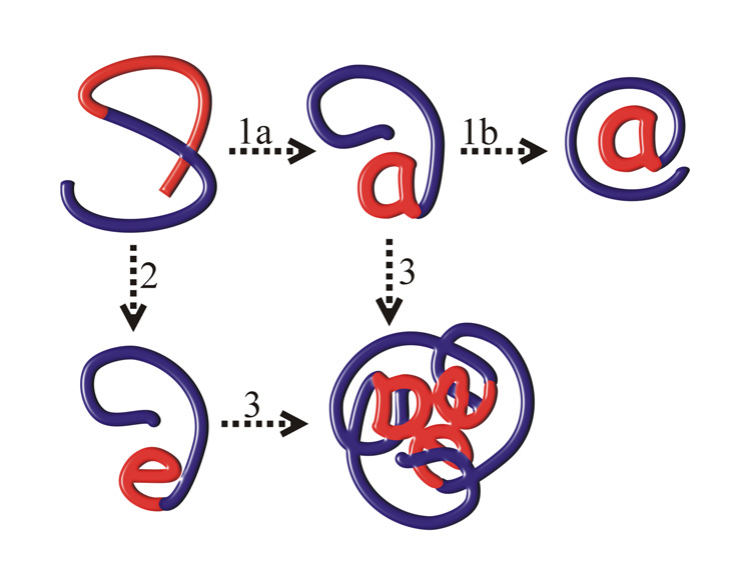
Simplified model of correct folding versus misfolding and aggregation. The correct protein folding pathway (1) often competes with misfolding (2) and aggregation (3). Aggregation occurs among intermediates with exposed hydrophobic patches, which are buried in the correctly folded protein (blue lines, hydrophilic solvent-exposed parts of the protein; red lines: hydrophobic patches).

In general, the methods used for inclusion body solubilization result in a soluble protein that is devoid of its native conformation. This protein must then be transferred into conditions that allow the formation of the native structure (*e.g*. low denaturant concentration). Moreover, appropriate redox conditions have to be established when the protein contains disulfide bonds in the native state. When proper conditions for refolding are identified, the refolding process can require a few seconds or several days. During this period, the correct refolding pathway competes, often in disadvantage, with misfolding and aggregation of the target protein (Figure 1). Protein refolding involves intramolecular interactions and follows first order kinetics [32-35]. Protein aggregation, however, involves intermolecular interactions and, thus, is a kinetic process of second or higher order, which is favored at high protein concentrations [32-35]. In fact, refolding yields commonly decrease with increasing initial concentrations of the unfolded protein independent of the refolding method applied [35-40].

Aggregates are formed by nonnative intermolecular hydrophobic interactions between protein folding intermediates, which have not yet buried their hydrophobic amino acid stretches (Figure 1). When the refolding process is beyond these aggregation-prone intermediates, the productive folding pathway is favored and aggregation does not occur. Therefore, prevention of hydrophobic intermolecular interaction during the first steps of refolding is crucial to allow successful renaturation at high protein concentrations. Only recently a non-empirical method for predicting the fate of proteins during the refolding process was proposed [41]. It is based on the second viral coefficient, which indicates the magnitude of protein interaction under certain refolding conditions, and thus its tendency to aggregate. However, though being soluble in the refolding buffer is essential for a protein molecule to refold, it does not ensure that it will fold into the native form.

#### Are further purification steps required after solubilization of inclusion bodies?

The recombinant target protein represents in general the major fraction of the inclusion body proteins. Therefore, refolding attempts can be undertaken directly after solubilization of the inclusion bodies. Some reports, however, claim higher refolding yields when the solubilized inclusion body proteins are purified prior to the refolding attempt [36,39,42,43]. Additional purification has been recommended when the protein of interest represents less than 2–5% of the total cell protein [26] or less than 2/3 of the total inclusion body protein [42]. The type of contaminants can also be crucial for the success of the refolding process. For example, typical non-proteinaceous contaminants of inclusion body preparations did not affect refolding yields of lysozyme, while proteinaceous contaminants, which have a high tendency towards aggregation significantly reduced refolding yields [42]. Further purification prior to the refolding attempt does not seem to be required, even at low target protein concentrations, when the solubilized inclusion body proteins are subjected to refolding conditions during size exclusion chromatography where refolding and purification can occur simultaneously [44]. All pros and cons of any further purification step have to be carefully evaluated as they cause potential protein loss and additional production costs.

### Techniques for protein refolding

#### Direct dilution

The simplest refolding procedure is to dilute the concentrated protein-denaturant solution into a refolding buffer that allows the formation of the native structure of the protein. Most frequently, the final protein concentration after dilution is in the 1–10 μg/ml range in order to favor the productive refolding instead of the unproductive aggregation pathway. Though ideal at laboratory scale, this technique has serious drawbacks during scale-up as huge refolding vessels and additional cost-intensive concentration steps are required after renaturation.

A major improvement of this technique was the development of a method where the solubilized, denatured protein is added in pulses or continuously into the refolding buffer [37,40,45-47]. This technique still keeps the simplicity of the direct dilution method while considerably increasing the final concentration of the refolded protein. Prerequisite is an appropriate knowledge of the folding kinetics of the target protein. The addition of the concentrated protein-denaturant solution should occur at rates slower than the rate-determining folding step of the target protein, thereby avoiding the accumulation of aggregation-prone folding intermediates [37,46]. For pulse addition it has been recommended that 80% of the maximum refolding yield should be reached before adding the next pulse [14]. Other factors to be considered are the increasing residual concentration of the denaturant with each pulse, which should not surpass concentrations that affect the refolding of the protein, and the amount of protein added per pulse, which should be optimized in batch experiments to minimize aggregation [14].

#### Membrane controlled denaturant removal

Another technique to transfer the solubilized and unfolded protein to conditions allowing the formation of the native structure is the utilization of dialysis and diafiltration systems for denaturant removal [*e.g*. [48-51]]. In contrast to the direct dilution method, the change from denaturing to native buffer conditions occurs gradually. Thus, the protein passes through different regimes of denaturant concentrations, where folding intermediates that are prone to aggregation may become populated. Most often, these techniques cause more aggregation during refolding compared to the direct dilution method [*e.g*. [52]]. Additionally, refolding yields can be negatively affected by non-specific adsorption of protein to the membrane. However, for some proteins and with the appropriate denaturant removal rates, adapted to the requirements of the target protein, high refolding yields at high protein concentrations can be obtained [50-53]. A fairly simple device was recently introduced allowing continuous or pulse refolding in a similar way as in the direct dilution method [54].

#### Chromatographic methods for protein refolding

##### Protein refolding based on size exclusion chromatography

Buffer exchange for denaturant removal can also be carried out by using size exclusion chromatography (SEC). Most frequently, the denaturant-protein solution is injected into a column previously equilibrated with the refolding buffer [44,55-58]. Subsequent elution with the refolding buffer results in a refolded protein in the eluate fraction with a considerably higher concentration compared to concentrations that can be reached by the simple dilution technique [44,56,58]. Protein refolding may be completed in the column or for proteins with slow folding kinetics the final folding steps may occur in the eluate fraction [44]. Aggregate formation is supposed to be reduced either by physical separation of aggregation-prone folding intermediates in the porous structures of the gel [56] or, more likely, by resolubilization of formed aggregates through the delayed running front of the denaturant, which gives the solubilized aggregates another opportunity to refold [14]. For proteins, which exhibit superior refolding yields during gradual denaturant removal, such as lysozyme, elution during SEC is preferably performed by using a decreasing denaturant gradient [38,52,59]. In specific cases, the denaturant removal can be accompanied with other changes in the buffer composition (*i.e*. pH) for further optimization of refolding conditions [52]. An additional advantage of this chromatographic method is the concomitant purification of the target protein during the refolding process [44]. Furthermore, some recent applications have shown the feasibility of using SEC for continuous processes of protein refolding [60,61]. Also, SEC in combination with the use of an annular chromatography system can be coupled to an ultrafiltration and recycling unit for reinjection of resolubilized aggregates, which may form during the refolding process [60].

Some parameters for refolding using SEC are of key importance. For example, protein aggregation during sample injection can cause low refolding yields [62]; injecting the sample followed by an additional small volume of denaturant solution solves this problem [44,52,62]. Also, optimum results can only be reached when the properties of the chromatographic resin allow efficient separation of the renatured target protein from different folding intermediates, misfolded protein, and aggregates that might form during the refolding process [59,63,64]. In general, lower refolding yields are obtained by injecting the denatured protein at high concentrations [38,58,60,61,64] and/or by elution at high rates [52,62,64]. Both conditions result in poor separation among different folding intermediates thereby boosting protein precipitation.

##### Matrix-assisted protein refolding

Attaching the solubilized and unfolded protein to a solid support prior to changing from denaturing to native buffer conditions is another approach to avoid the unwanted intermolecular interaction between aggregation-prone folding intermediates. Binding of the solubilized and unfolded protein to the matrix requires the formation of a stable protein-matrix complex withstanding the presence of chaotropic agents. However, after changing to native buffer conditions, the detachment of the refolded target protein from the matrix should easily be accomplished. Several combinations of binding motives and matrices have been employed for binding the unfolded protein to the solid support. For example, proteins with a natural occurring charged patch in the unfolded chain, which binds to ion exchange resins [59,65-67], or proteins containing artificially engineered peptide tags such as the hexahistidine tag, which binds to immobilized metal ions [59,68-70], or N- or C-terminal hexaarginine tags binding to a polyanionic support [71], or protein fusions with denaturant-resistant binding domains, such as a glutathione S-transferase fragment, which binds to an anion exchange matrix [72] or the cellulose binding domain of the cellulose degrading multienzyme complex of the thermophilic bacterium *Clostridium thermocellum*, which binds to a cellulose matrix [73], have been employed. After binding, the matrix-protein complex is brought to refolding conditions by any of the above-mentioned techniques such as dilution [71], dialysis [68,73], or buffer exchange through chromatography [59,66,69,70,72]. Finally, the refolded protein can be detached from the matrix, *e.g*. in the case hexahistidine-tagged proteins by elution with EDTA [69] or imidazole [59,70] or by buffers with high ionic strength in the case of proteins bound by ionic interactions [59,65,66,71,72]. Due to the selective binding, matrix-assisted refolding can combine the renaturation of the target protein along with its purification from host cell protein contaminants [69,70,72].

##### Refolding using hydrophobic interaction chromatography

Hydrophobic interaction chromatography (HIC) has also been successfully used for protein refolding with concomitant removal of contaminating proteins during the renaturation process [74-78]. Unfolded proteins are applied to the column at high salt concentrations and refolded and eluted with a decreasing salt gradient. In contrast to the above-mentioned chromatographic methods there is no requirement for typical refolding aiding agents such as arginine during the in-column refolding process. Moreover, refolding of the disulfide containing protein proinsulin was even obtained in the absence of a redox system in the mobile phase [76].

It has been proposed that refolding is facilitated during HIC because unfolded proteins adsorb at high salt concentrations to the hydrophobic matrix and, thus, are not prone to aggregation. Additionally, hydrophobic regions of the protein that adsorb to the HIC matrix form microdomains around which native structure elements can form. During migration through the column, the protein will pass through several steps of adsorption and desorption, controlled by the salt concentration and hydrophobicity of the intermediate(s), resulting finally in the formation of the native structure [75].

### Physical and chemical features improving protein refolding yields

Apart from any of the above-mentioned techniques for protein refolding, there are physical and chemical variables that have a great impact on the final yield of biologically active protein. For example, temperature as well as the composition of the refolding buffer are important variables influencing the final refolding yield.

#### Physical variables aiding protein refolding

The most important physical variable influencing the refolding yield is the temperature [40,45,50,51]. Temperature has a dual effect on the refolding process. On one side, it influences the speed of folding and on the other it influences the propensity towards aggregation of folding intermediates with exposed hydrophobic patches. Also, there is limited temperature range in which each protein is thermodynamically stable in a given buffer system [79]. In general, low temperatures support the productive folding pathway as hydrophobic aggregation is suppressed. However, low temperatures also slow down the folding rates, thus increasing the time required for renaturation [51]. For refolding attempts of a new protein, 15°C has been proposed as a good starting point [14].

Pressure was identified as another important physical variable affecting protein structure as well as protein refolding processes [80]. It was shown that high pressure up to 3 kbar can disrupt oligomeric protein structures [80] and can dissolve protein aggregates and inclusion bodies [81,82]. The disassembled protein monomers retain native-like secondary structure up to 5 kbar [80]. After gradual depressurization, they can reach their native state even at high protein concentrations, because folding intermediates prone to aggregate at atmospheric pressure are prevented from aggregation by high pressure [81-83].

#### Chemicals aiding protein refolding

Certainly, L-arginine is nowadays the most commonly used refolding aiding agent [14]. It impedes aggregate formation by enhancing the solubility of folding intermediates, presumably by shielding hydrophobic regions of partially folded chains. In addition, it has been shown that numerous other low molecular weight additives such as detergents, protein-stabilizing agents such as glycerol or even low residual concentrations of denaturants improve refolding yields by suppressing aggregation [14]. In addition, high-molecular weight additives such as polyethylene glycol were used successfully for enhancing protein refolding yields [84]. More recently, low-molecular weight non-detergent zwitterionic agents such as sulfobetaines, substituted pyridines and pyrroles and acid substituted aminocyclohexanes have been employed successfully for protein renaturation [40,85-87]. Moreover, polymers with temperature-dependent hydrophobicity were effectively applied for protein refolding at higher temperatures [88,89]. The benefit of each of these refolding aiding agents for a given renaturation system has to be elucidated experimentally, as they are not equally advantageous for all proteins. The mechanisms of interactions of these refolding aiding agents with the folding intermediates remain often obscure although it is clear that all these substances suppress aggregation in favor of the productive folding pathway [90].

#### Micelles and liposomes as protein refolding aiding systems

Detergents [91,92] and phospholipids [93,94], in the form of micelles and liposomes [95], respectively, as well as mixed micelle systems formed by phospholipids and detergents [92,95,96] have shown potential to aid protein refolding. Most likely, illegitimate hydrophobic interactions between folding intermediates are suppressed by transient nonpolar interactions between the protein and the micelle or liposome [91-93]. Additional transient polar interactions in mixed micelles are supposed to be responsible for higher refolding yields compared to only detergent-based micelle systems [92,96]. Moreover, liposomes linked covalently to chromatographic resins have potential to combine renaturation and separation of the refolded target protein [93,94].

Reversed micelles, formed when an aqueous detergent solution is mixed with an organic solvent, can also facilitate protein refolding by avoiding aggregate formation [97]. The denatured protein, once transferred to this solution, tries to avoid the organic phase, and, after reaching the hydrophilic core of the reversed micelle, can refold as a single molecule [97]. Recently, it was demonstrated that protein precipitates can be solubilized by direct addition into the reversed micellar system allowing refolding with high yields at high protein concentrations [98-100]. Yet, direct solubilization of inclusion bodies in reversed micellar systems has not been reported. In addition, recovery of refolded protein from these micellar structures is not easily accomplished [97,99].

### Chemical and biological protein refolding aiding agents mimicking *in vivo *folding conditions

#### Natural chaperones

Chaperones are a group of proteins conserved in all kingdoms, which play a key role in assisting *in vivo *protein folding and protecting cellular proteins from different types of environmental stress by suppressing protein aggregation. For example, the major *E. coli *chaperonin GroEL is involved in the *in vivo *folding of 10% of all newly synthesized proteins at normal growing conditions, and of 30% under stress conditions [101]. GroEL assists protein folding by a first capturing step of aggregation-prone folding intermediates [102]. The release of the folding-competent form is then accomplished in an ATP-dependent fashion through the action of the cochaperonin GroES [102].

Natural chaperones have also been applied successfully to refold various proteins *in vitro *[103]. However, their routine application is limited by their cost, the relatively high chaperone concentration required (at least equimolar to the target protein) and the need for their removal after the refolding procedure [103,104]. Some procedures have tried to overcome these limitations by utilizing immobilized and reusable (mini)-chaperone systems [104-106]. Nevertheless, chaperone-based refolding processes are not robust enough for large-scale processes [14].

#### Artificial chaperones

A further development of the detergent-based micellar system mimics the two-step mechanism of chaperone-assisted protein folding. The capturing step is performed by diluting the denatured protein into a detergent solution, which prevents protein aggregation through the formation of mixed protein-detergent micelles [107-110]. Aqueous solutions of hydrogel nanoparticles (*e.g*. self-assembly of hydrophobized polysaccharides such as cholesterol-bearing pullulan) have been also used for the capturing step [111]. The release of the folding-competent protein is subsequently initiated by the addition of cyclodextrins [107-112]. They are added in excess to the capturing agent and strip the detergent from the protein-detergent micelles through the formation of a tight detergent-cyclodextrin complex. Long cyclodextrin polymers as striping agent were reported to result in higher refolding yields compared to monomeric cyclodextrins [113]. Also, rapid addition of soluble cyclodextrins is thought to result in higher refolding yields compared to slow addition [108,109] or the utilization of immobilized cyclodextrins [108,109]. However, at least for α-glucosidase similar refolding yields were reported by stripping the detergent either with soluble or immobilized cyclodextrins [114]. The utilization of these cyclodextrin polymer beads allows simple removal of the cyclodextrin-detergent complex by centrifugation and, moreover, these beads can be used in expanded-bed columns in semicontinuous refolding processes [114].

#### Liquid paraffin as pseudolipid bilayer membrane

*In vivo*, many proteins are transported through bilayered membranes in an extended and partially unfolded form either simultaneously or after their synthesis [115]. A rather peculiar protein refolding procedure mimicking the effect of a bilayered membrane was carried out in a three-phase liquid system built up in a centrifugation tube [116]. The upper phase contained an organic solution, which was separated from the aqueous refolding buffer by liquid paraffin. The protein, in an aggregated and denatured form, was added to the organic phase and forced to pass through the paraffin film into the refolding buffer by centrifugation. This procedure was successfully applied for the refolding of aggregated and denatured preparations of the model proteins RNase A and BSA.

#### Template-assisted protein folding

Several proteins are synthesized in their natural environment with amino-terminal propeptides usually located between a signal sequence and the mature part of the protein. *In vivo*, these propeptides are known to play a key role in assisting the correct folding of the mature part of the protein [117]. *In vitro *studies have demonstrated that they can facilitate the refolding in *cis*, when the denatured mature protein is still linked to its propeptide prior to the transfer into the refolding buffer, or in *trans *by including the isolated propeptide into the refolding buffer [118-120]. This propeptide assisted protein refolding can be exploited for the renaturation of inclusion body proteins either by synthesizing the mature part linked to its propeptide, thus allowing later facilitated refolding [121,122], or by synthesizing the mature protein and then including the appropriate propeptide into the refolding buffer [123].

Another method of template-assisted protein refolding exploits the specific binding properties of monoclonal antibodies to the target protein to reduce the time required for protein refolding and to enhance the final refolding yield [124,125]. This procedure does not work with all antibodies and depends on the availability of specific antibody clones. Thus, it represents more a proof-of-principle rather than a practical approach to generate active proteins through refolding of inclusion body proteins.

### Proteins containing disulfide bonds: special requirements

In general, solubilization of inclusion body proteins by chaotropic agents is carried out in the presence of reducing agents such as dithiothreitol or β-mercaptoethanol to allow the disruption of nonnative disulfide bonds. Following solubilization, naturally disulfide-bonded proteins have to be refolded under conditions, which permit the formation of their native disulfide bonds. In the simplest way, free cysteine residues can be oxidized by molecular oxygen, a redox reaction catalyzed by Cu^2+ ^ions [126,127]. Though a cheap option, air oxidation is slow, often results in mismatched disulfides, and is not suitable for disulfide-bonded proteins, which also have free cysteines [26].

Disulfide bonds are more efficiently formed when a mixture of low molecular weight thiols (*e.g*. glutathione) in their reduced and oxidized state is added to the refolding buffer [126,128]. Best conditions for refolding of disulfide-bonded proteins are commonly established when the reduced form is present in excess and the pH is slightly alkaline. These conditions allow rapid disulfide exchange reactions until the protein reaches the most stable disulfide-bonded configuration, in general the native state of the protein [26,126,128-130]. Recently, a novel generation of aromatic thiols was developed which have lower p*K*_a _values as the aliphatic thiols thus enabling disulfide-bond formation at lower pH values [131,132]. These thiol reagents might be useful for the refolding of proteins with limited stability at alkaline conditions. Also, an immobilized disulfide-reshuffling system based on thiol-carrying latex particles has recently been successfully applied for the refolding of RNase A [133,134].

Naturally disulfide-bonded proteins in their reduced states are often very unstable and exhibit a high tendency towards aggregation, especially during the early stages of refolding [128]. These problems can be overcome by modifying the reduced thiol groups in the unfolded protein, either by S-sulfonation [39,128,135,136] or by transforming the free cysteines into mixed disulfides with the oxidized form of a thiol reagent (*e.g*. glutathione) [128,137]. These chemical modifications introduce numerous charged residues into the protein, which prevent the intermolecular interactions responsible for aggregation. The chemically modified protein is then transferred to refolding conditions. Correct disulfide bond formation for S-sulfonated proteins is initiated by supplementing the refolding buffer with the appropriate redox system [39,128,135,136], or, for proteins with mixed disulfides by adding trace amounts of the reduced form of the thiol reagent [128,137].

Improvements of refolding yields of disulfide-bonded proteins have also been achieved by using protein disulfide isomerase (PDI) in combination with a redox system. PDI is a folding catalyst that assists disulfide bond formation *in vivo *[138] and was successfully implemented for aiding disulfide bond formation during *in vitro *protein refolding [139,140]. In some cases PDI did not show significant effects on the refolding yield but significantly increased the refolding rate [141]. However, residual concentrations of chaotropic agents in the refolding buffer, especially guanidinium hydrochloride, can drastically reduce PDI activity [142]. Traces of small peptides containing the active site of PDI [143] and chemically synthesized dithiol molecules mimicking PDI function [144-146] have also shown potential to increase the *in vitro *refolding yields generally obtained with the common redox systems [143,146].

### Process integration

Published refolding processes are often composed of numerous and cumbersome steps, both downstream (*e.g*. cell disruption, inclusion body isolation and purification by several centrifugation and washing steps followed by a final solubilization procedure) and upstream of the renaturation process (*e.g*. removal of aggregates and misfolded protein and final purification of the correctly refolded target protein). Scale-up problems can arise when some of these steps are not transferable to larger scale processes. As an alternative to the common downstream process, inclusion bodies can directly be solubilized from chemically treated *E. coli *cells [147-150] or in combination with mechanical treatments [72]. Even more, inclusion body solubilization directly from cells in the cultivation broth is feasible as was shown for periplasmic [147] as well as for cytoplasmic inclusion bodies [151]. A high degree of purification, removal of cell debris and *E. coli *host cell proteins, can be achieved by selective extraction of inclusion body proteins combined with diafiltration [148], aqueous two-phase extraction [147] or selective capture by either expanded bed chromatography [72,149,151] or by attachment to magnetic particles recoverable in high gradient magnetic fields [152]. Major difficulties often arise by the increase of broth viscosity due to release of DNA after chemical treatment requiring its selective removal *e.g*. by precipitation through spermidine addition [149,151] or preferably by treatment with DNA-degrading enzymes [153]. Afterwards, the prepurified and solubilized target protein can be subjected to refolding conditions using any of the above-mentioned methods. Moreover, there are reports on integrated processes where solubilization of the target protein from chemically treated cells is followed by a chromatographic process in which the capturing step and removal of *E. coli *contaminants is followed directly and in the same operation unit by refolding and subsequent purification [72,74]. Utilization of refolding methods based on chromatographic processes is additionally advantageous as they combine refolding with an at least partial purification of the target protein [44,69-72]. In addition, aggregates formed during the refolding process can also be removed through chromatographic processes as they have a different retention time compared to the correctly folded protein [56,64,67]. Finally, chromatographic processes can be performed continuously [60,61] with the possibility to recycle aggregates formed during the refolding process thus leading to processes with refolding yields up to 100% [60].

## Perspectives

After the first enthusiasm about protein production using recombinant microorganisms, it was promptly understood that obtaining an active form of the desired protein was not a simple task. Many proteins form nonnative precipitates in form of inclusion bodies when synthesized in bacteria and there is no universal refolding recipe for the generation of native protein from solubilized inclusion bodies. For any given protein, the best refolding conditions still have to be determined empirically. Among a lot of experience and "a good feeling for the best way", the use of experimental design methodologies [154,155] and further improvements in predicting the likelihood of aggregation [41,156] may increase the speed for finding the optimal refolding conditions for a given protein. Also, less-established and new techniques as well as new refolding aiding additives may become more widely used in the near future. However, these techniques or new protein refolding aiding substances await rigorous testing for refolding of not only easy-going model proteins such as RNase A but also for more recalcitrant inclusion body proteins. Moreover, refolding strategies also have to be adapted to the required quantity and final use of the refolded protein. For therapeutic proteins needed in great quantities more effort can be undertaken to identify the best refolding conditions leading to high yields of the correctly folded protein. For a protein where just a few milligrams are required for biochemical and/or structural studies process optimization with respect to high yields is not such a necessity. Also, special demands for high throughput refolding screening arising from structural genomic projects require robust strategies that will lead to monodisperse refolded protein samples [157]. In this case, the direct dilution method in combination with variations in temperature and buffer composition is still the best approach. Altogether, new strategies need to increase the robustness of refolding processes and/or decrease the costs to find acceptance for broader applications.

## References

[B1] Carrió MM, Villaverde A (2001). Protein aggregation as bacterial inclusion bodies is reversible. FEBS Lett.

[B2] Datar RV, Cartwright T, Rosen C-G (1993). Process economics of animal cell and bacterial fermentations: A case study analysis of tissue plasminogen activator. Bio/Technology.

[B3] Qiu J, Swartz JR, Georgiou G (1998). Expression of active human tissue-type plasminogen activator in *Escherichia coli*. Appl Environ Microbiol.

[B4] Zhan X, Schwaller M, Gilbert HF, Georgiou G (1999). Facilitating the formation of disulfide bonds in the *Escherichia coli *periplasm via coexpression of yeast protein disulfide isomerase. Biotechnol Prog.

[B5] Schäffner J, Winter J, Rudolph R, Schwarz E (2001). Cosecretion of chaperones and low-molecular-size medium additives increases the yield of recombinant disulfide-bridged proteins. Appl Environ Microbiol.

[B6] Manosroi J, Tayapiwatana C, Götz F, Werner RG, Manosroi A (2001). Secretion of active recombinant human tissue plasminogen activator derivatives in *Escherichia coli*. Appl Environ Microbiol.

[B7] Manosroi J, Tayapiwatana C, Manosroi A, Beer J, Bergemann K, Werner RG (2002). Lekteplase – a secreted tissue plasminogen activator derivative from *Escherichia coli*. Arzneim-Forsch.

[B8] Martegani E, Forlani N, Mauri I, Porro D, Schleuning WD, Alberghina L (1992). Expression of high levels of human tissue plasminogen activator in yeast under the control of an inducible GAL promoter. Appl Microbiol Biotechnol.

[B9] Wiebe MG, Karandikar A, Robson GD, Trinci APJ, Flores Candia J-L, Trappe S, Wallis G, Rinas U, Derkx PMF, Madrid SM, Sisniega H, Faus I, Montijn R, van den Hondel CAMJJ, Punt PJ (2001). Production of tissue plasminogen activator (t-PA) in *Aspergillus nig*er. Biotechnol Bioeng.

[B10] Farrell PJ, Behie LA, Iatrou K (1999). Transformed lepidopteran insect cells: New sources of recombinant human tissue plasminogen activator. Biotechnol Bioeng.

[B11] Lilie H, Schwarz E, Rudolph R (1998). Advances in refolding of proteins produced in *E. coli*. Curr Opin Biotechnol.

[B12] De Bernardez Clark E (1998). Refolding of recombinant proteins. Curr Opin Biotechnol.

[B13] Gergiou G, Valax P (1999). Isolating inclusion bodies from bacteria. Methods Enzymol.

[B14] De Bernardez Clark E, Schwarz E, Rudolph R (1999). Inhibition of aggregation side reactions during in vitro protein folding. Methods Enzymol.

[B15] De Bernardez Clark E (2001). Protein refolding for industrial processes. Curr Opin Biotechnol.

[B16] Middelberg APJ (2002). Preparative protein refolding. Trends Biotechnol.

[B17] Tsumoto K, Ejima D, Kumagai I, Arakawa T (2003). Practical considerations in refolding proteins from inclusion bodies. Protein Expr Purif.

[B18] Fahnert B, Lilie H, Neubauer P (2004). Inclusion bodies: Formation and utilization. Adv Biochem Eng/Biotechnol.

[B19] Rinas U, Bailey JE (1992). Protein compositional analysis of inclusion bodies produced in recombinant *Escherichia coli*. Appl Microbiol Biotechnol.

[B20] Hagel P, Gerding JJT, Fieggen W, Bloemendal H (1971). Cyanate formation in solutions of urea I. Calculation of cyanate concentrations at different temperature and pH. Biochim Biophys Acta.

[B21] Cejka J, Vodražka Z, Salák J (1968). Carbamylation of globin in electrophoresis and chromatography in the presence of urea. Biochim Biophys Acta.

[B22] Estapé D, Rinas U (1996). Optimized procedures for purification and solubilization of basic fibroblast growth factor inclusion bodies. Biotechnol Tech.

[B23] Iwakura M, Obara K, Kokubu T, Ohashi S, Izutsu H (1992). Expression and purification of growth hormone-releasing factor with the aid of dihydrofolate reductase handle. J Biochem (Tokyo).

[B24] Khan RH, Appa Rao KBC, Eshwari ANS, Totey SM, Panda AK (1998). Solubilization of recombinant ovine growth hormone with retention of native-like secondary structure and its refolding from the inclusion bodies of *Escherichia coli*. Biotechnol Prog.

[B25] Patra AK, Mukhopadhyay R, Mukhija R, Krishnan A, Garg LC, Panda AK (2000). Optimization of inclusion body solubilization and renaturation of recombinant human growth hormone from *Escherichia coli*. Protein Expr Purif.

[B26] Thatcher DR, Hitchcock A, Pain HR (1994). Protein folding in biotechnology. In Mechanisms of protein refolding.

[B27] Puri NK, Crivelli E, Cardamone M, Fiddes R, Bertolini J, Ninham B, Brandon MR (1992). Solubilization of growth hormone and other recombinant proteins from *Escherichia coli *by using a cationic surfactant. Biochem J.

[B28] Kurucz I, Titus JA, Jost CR, Segal DM (1995). Correct disulfide pairing and efficient refolding of detergent-solubilized single-chain Fv proteins from bacterial inclusion bodies. Mol Immunol.

[B29] Tsumoto K, Umetsu M, Kumagai I, Ejima D, Arakawa T (2003). Solubilization of active green fluorescent protein from insoluble particles by guanidine and arginine. Biochem Biophys Res Commun.

[B30] Umetsu M, Tsumoto K, Ashish K, Nitta S, Tanaka Y, Adschiri T, Kumagai I (2004). Structural characteristics and refolding of in vivo aggregated hyperthermophilic archaeon proteins. FEBS Lett.

[B31] Tokatlidis K, Dhurjati P, Millet J, Béguin P, Aubert J-P (1991). High activity of inclusion bodies formed in *Escherichia coli *overproducing *Clostridium thermocellum *endoglucanase D. FEBS Lett.

[B32] Zettlmeissl G, Rudolph R, Jaenicke R (1979). Reconstitution of lactic dehydrogenase. Noncovalent aggregation vs. reactivation. 1. Physical properties and kinetics of aggregation. Biochemistry.

[B33] Kiefhaber T, Rudolph R, Kohler H-H, Buchner J (1991). Protein aggregation in vitro and in vivo: A quantitative model of the kinetic competition between folding and aggregation. Bio/Technology.

[B34] Goldberg ME, Rudolph R, Jaenicke R (1991). A kinetic study of the competition between renaturation and aggregation during the refolding of denatured-reduced egg white lysozyme. Biochemistry.

[B35] De Bernardez Clark E, Hevehan D, Szela S, Maachupalli-Reddy J (1998). Oxidative renaturation of hen egg-white lysozyme. Folding vs aggregation. Biotechnol Prog.

[B36] Tran-Moseman A, Schauer N, De Bernardez Clark E (1999). Renaturation of *Escherichia coli*-derived recombinant human macrophage colony-stimulating factor. Protein Expr Purif.

[B37] Katoh S, Katoh Y (2000). Continous refolding of lysozyme with fed-batch addition of denatured protein solution. Process Biochem.

[B38] Gu Z, Su Z, Janson J-C (2001). Urea gradient size-exclusion chromatography enhanced the yield of lysozyme refolding. J Chromatogr A.

[B39] Tikhonov RV, Pechenov SE, Belacheu IA, Yakimov SA, Klyushnichenko VE, Tunes H, Thiemann JE, Vilela L, Wulfson AN (2002). Recombinant human insulin IX. Investigation of factors, influencing the folding of fusion protein-S-sulfonates, biotechnological precursors of human insulin. Protein Express Purif.

[B40] Vallejo LF, Rinas U (2004). Optimized procedure for renaturation of recombinant human bone morphogenetic protein-2 at high protein concentration. Biotechnol Bioeng.

[B41] Ho JGS, Middelberg APJ, Ramage P, Kocher HP (2003). The likelihood of aggregation during protein renaturation can be assessed using the second virial coefficient. Protein Sci.

[B42] Maachupalli-Reddy J, Kelley BD, De Bernardez Clark E (1997). Effect of inclusion body contaminants on the oxidative renaturation of hen egg white lysozyme. Biotechnol Prog.

[B43] Batas B, Schiraldi C, Chaudhuri JB (1999). Inclusion body purification and protein refolding using microfiltration and size exclusion chromatography. J Biotechnol.

[B44] Müller C, Rinas U (1999). Renaturation of heterodimeric platelet-derived growth factor from inclusion bodies of recombinant *Escherichia coli *using size-exclusion chromatography. J Chromatogr A.

[B45] Buchner J, Pastan I, Brinkmann U (1992). A method for increasing the yield of properly folded recombinant fusion proteins: Single-chain immunotoxins from renaturation of bacterial inclusion bodies. Anal Biochem.

[B46] Fischer B, Perry B, Sumner I, Goodenough P (1992). A novel sequential procedure to enhance the renaturation of recombinant protein from *Escherichia coli *inclusion bodies. Protein Eng.

[B47] Terashima M, Suzuki K, Katoh S (1996). Effective refolding of fully reduced lysozyme with a flow-type reactor. Process Biochem.

[B48] Maeda Y, Koga H, Yamada H, Ueda T, Imoto T (1995). Effective renaturation of reduced lysozyme by gentle removal of urea. Protein Eng.

[B49] Varnerin JP, Smith T, Rosenblum CI, Vongs A, Murphy BA, Nunes C, Mellin TN, King JJ, Burgess BW, Junker B, Chou M, Hey P, Frazier E, MacIntyre DE, van der Ploeg LHT, Tota MR (1998). Production of leptin in *Escherichia coli*: A comparison of methods. Protein Expr Purif.

[B50] West SM, Chaudhuri JB, Howell JA (1998). Improved protein refolding using hollow-fibre membrane dialysis. Biotechnol Bioeng.

[B51] Yoshii H, Furuta T, Yonehara T, Ito D, Linko Y-Y, Linko P (2000). Refolding of denatured/reduced lysozyme at high concentration with diafiltration. Biosci Biotechnol Biochem.

[B52] Gu Z, Weidenhaupt M, Ivanova N, Pavlov M, Xu B, Su Z-G, Janson J-C (2002). Chromatographic methods for the isolation of, and refolding of proteins from, *Escherichia coli *inclusion bodies. Protein Express Purif.

[B53] Horowitz PM, Simon D (1986). The enzyme rhodanese can be reactivated after denaturation in guanidium chloride. J Biol Chem.

[B54] Sørensen HP, Sperling-Petersen HU, Mortensen KK (2003). Dialysis strategies for protein refolding: Preparative streptavidin production. Protein Express Purif.

[B55] Werner MH, Clore GM, Gronenborn AM, Kondoh A, Fisher RJ (1994). Refolding proteins by gel filtration chromatography. FEBS Lett.

[B56] Batas B, Chaudhuri JB (1996). Protein refolding at high concentration using size-exclusion chromatography. Biotechnol Bioeng.

[B57] Batas B, Chaudhuri JB (1999). Considerations of sample application and elution during size-exclusion chromatography-based protein refolding. J Chromatogr A.

[B58] Fahey EM, Chaudhuri JB (2000). Refolding of low molecular weight urokinase plasminogen activator by dilution and size exclusion chromatography-a comparative study. Sep Sci Technol.

[B59] Li M, Poliakov A, Danielson UH, Su Z, Janson JC (2003). Refolding of a recombinant full-length non-structural (NS3) protein from hepatitis C virus by chromatographic procedures. Biotechnol Lett.

[B60] Schlegl R, Iberer G, Machold C, Necina R, Jungbauer A (2003). Continuous matrix-assisted refolding of proteins. J Chromatogr A.

[B61] Lanckriet H, Middelberg APJ (2004). Continuous chromatographic protein refolding. J Chromatogr A.

[B62] Liu H-S, Chang C-K (2003). Chaperon solvent plug to enhance protein refolding in size exclusion chromatography. Enzyme Microb Technol.

[B63] Fahey EM, Chaudhuri JB, Binding P (2000). Refolding and purification of a urokinase plasminogen activator fragment by chromatography. J Chromatogr B.

[B64] Gu Z, Zhu X, Ni S, Zhou H, Su Z (2003). Inhibition of aggregation by media selection, sample loading and elution in size exclusion chromatographic refolding of denatured bovine carbonic anhydrase B. J Biochem Biophys Methods.

[B65] Suttnar J, Dyr JE, Hamšíková E, Novák J, Vonka V (1994). Procedure for refolding and purification of recombinant proteins from *Escherichia coli *inclusion bodies using a strong anion exchanger. J Chromatogr B.

[B66] Creighton TE (1986). Folding of proteins adsorbed reversibly to ion-exchange resins. UCLA Symp Mol Cell Biol.

[B67] Li M, Zhang G, Su Z (2002). Dual gradient ion-exchange chromatography improved refolding yield of lysozyme. J Chromatogr A.

[B68] Negro A, Onisto M, Grassato L, Caenazzo C, Garbisa S (1997). Recombinant human TIMP-3 from *Escherichia coli*: Synthesis, refolding physico-chemical and functional insights. Protein Eng.

[B69] Zouhar J, Nanak E, Brzobohatý B (1999). Expression, single-step purification, and matrix-assisted refolding of a maize cytokinin glucoside-specific β-glucosidase. Protein Expr Purif.

[B70] Rehm BHA, Qi Q, Beermann BB, Hinz H-J, Steinbüchel A (2001). Matrix assisted in vitro refolding of Pseudomonas aeruginosa class II polyhydroxylalkanoate synthase from inclusion bodies produced in recombinant *Escherichia coli*. Biochem J.

[B71] Stempfer G, Höll-Neugebauer B, Rudolph R (1996). Improved refolding of an immobilized fusion protein. Nature Biotechnol.

[B72] Cho TH, Ahn SJ, Lee EK (2001). Refolding of protein inclusion bodies directly from *E. coli *homogenate using expanded bed adsorption chromatography. Bioseparation.

[B73] Berdichevsky Y, Lamed R, Frenkel D, Gophna U, Bayer EA, Yaron S, Shoham Y, Benhar I (1999). Matrix-assisted refolding of single-chain Fv-cellulose binding domain fusion proteins. Protein Expr Purif.

[B74] Geng X, Chang X (1992). High-performance hydrophobic interaction chromatography as a tool for protein refolding. J Chromatogr A.

[B75] Geng X, Quan B (2002). Mechanism of simultaneously refolding and purification of proteins by hydrophobic interaction chromatographic unit and aplications. Sci China B.

[B76] Bai Q, Kong Y, Geng X (2003). Studies on renaturation with simultaneous purification of recombinant human proinsulin from *E. coli *with high perfomance hydrophobic interaction chromatography. J Liq Chromatogr R T.

[B77] Gong B, Wang L, Wang C, Geng X (2004). Preparation of hydrophobic interaction chromatographic packings based on monodisperse poly(glycidylmethacrylate-*co*-ethylenedimethacrylate) beads and their application. J Chromatogr A.

[B78] Wang C, Geng X, Wang D, Tian B (2004). Purification of recombinant bovine normal prion protein PrP(104–242) by HPHIC. J Chromatogr B.

[B79] Privalov PL, Creighton TE (1992). Physical basis of the stability of the folded conformations of proteins. In Protein folding.

[B80] Robinson CR, Sligar SG (1995). Hydrostatic and osmotic pressure as tools to study macromolecular recognition. Methods Enzymol.

[B81] Foguel D, Robinson CR, de Sousa PC, Silva JL, Robinson AS (1999). Hydrostatic pressure rescues native protein from aggregates. Biotechnol Bioeng.

[B82] St John RJ, Carpenter JF, Randolph TW (1999). High pressure fosters protein refolding from aggregates at high concentrations. Proc Natl Acad Sci USA.

[B83] St John RJ, Carpenter JF, Balny C, Randolph TW (2001). High pressure refolding of recombinant human growth hormone from insoluble aggregates: Structural transformations, kinetic barriers and energetics. J Biol Chem.

[B84] Cleland JL, Wang DIC (1990). Cosolvent assisted protein refolding. Bio/Technology.

[B85] Goldberg ME, Expert-Bezançon N, Vuillard L, Rabilloud T (1995). Non-detergent sulphobetaines: A new class of molecules that facilitate in vitro renaturation. Folding & Design.

[B86] Vicik SM Methods of refolding proteins by use of zwitterionic low molecular weight agents.

[B87] Vallejo LF, Brokelmann M, Marten S, Trappe S, Cabrera-Crespo J, Hoffmann A, Gross G, Weich HA, Rinas U (2002). Renaturation and purification of bone morphogenetic protein-2 produced as inclusion bodies in high-cell-density cultures of recombinant *Escherichia coli*. J Biotechnol.

[B88] Kuboi R, Morita S, Ota H, Umakoshi H (2000). Protein refolding using stimuli-responsive polymer-modified aqueous two-phase systems. J Chromatogr B.

[B89] Yoshimoto N, Hashimoto T, Felix MM, Umakoshi H, Kuboi R (2003). Artificial chaperone-assisted refolding of bovine carbonic anhydrase using molecular assemblies of stimuli-responsive polymers. Biomacromolecules.

[B90] Arakawa T, Tsumoto K (2003). The effects of arginine on refolding of aggregated proteins: Not facilitate refolding, but suppress aggregation. Biochem Biophys Res Commun.

[B91] Tandon S, Horowitz PM (1987). Detergent-assisted refolding of guanidium chloride-denatured rhodanese. J Biol Chem.

[B92] Zardeneta G, Horowitz PM (1992). Micelle-assisted protein folding. J Biol Chem.

[B93] Yoshimoto M, Kuboi R (1999). Oxidative refolding of denatured/reduced lysozyme utilizing the chaperone-like function of liposomes and immobilized liposome chromatography. Biotechnol Prog.

[B94] Yoshimoto M, Shimanouchi T, Umakoshi H, Kuboi R (2000). Immobilized liposome chromatography for refolding and purification of protein. J Chromatogr B.

[B95] Zardeneta G, Horowitz PM (1994). Detergent, liposome, and micelle-assisted protein refolding. Anal Biochem.

[B96] Zardeneta G, Horowitz PM (1994). Protein refolding at high concentrations using detergent/phospholipid mixtures. Anal Biochem.

[B97] Hagen AJ, Hatton TA, Wang DIC (1990). Protein refolding in reversed micelles. Biotechnol Bioeng.

[B98] Hashimoto Y, Ono T, Goto M, Hatton TA (1998). Protein refolding by reversed micelles utilizing solid-liquid extraction technique. Biotechnol Bioeng.

[B99] Goto M, Hashimoto Y, Fujita T-A, Ono T, Furusaki S (2000). Important parameters affecting efficiency of protein refolding by reversed micelles. Biotechnol Prog.

[B100] Vinogradov AA, Kudryashova EV, Levashov AV, van Dongen WMAM (2003). Solubilization and refolding of inclusion body proteins in reverse micelles. Anal Biochem.

[B101] Ewalt KL, Hendrick JP, Houry WA, Hartl FU (1997). In vivo observation of polypeptide flux through the bacterial chaperonin system. Cell.

[B102] Hartl FU (1996). Molecular chaperones in cellular protein folding. Nature.

[B103] Thomas JG, Ayling A, Baneyx F (1997). Molecular chaperones, folding catalysts, and the recoverey of active recombinant proteins from *E. coli*. Appl Biochem Biotechnol.

[B104] Buchner J, Brinkmann U, Pastan I (1992). Renaturation of a single-chain immunotoxin facilitated by chaperones and protein disulfide isomerase. Bio/Technology.

[B105] Altamirano MM, Golbik R, Zahn R, Buckle AM, Fersht AR (1997). Refolding chromatography with immobilized mini-chaperones. Proc Natl Acad Sci USA.

[B106] Gao Y-G, Guan Y-X, Yao S-J, Cho M-G (2003). On-column refolding of recombinant human interferon-γ with an immobilized chaperone fragment. Biotechnol Prog.

[B107] Rozema D, Gellman SH (1995). Artificial chaperones: Protein refolding via sequential use of detergent and cyclodextrin. J Am Chem Soc.

[B108] Rozema D, Gellman SH (1996). Artificial chaperone-assisted refolding of carbonic anhydrase B*. J Biol Chem.

[B109] Rozema D, Gellman SH (1996). Artificial chaperone-assisted refolding of denatured-reduced lysozyme: Modulation of the competition between renaturation and aggregation. Biochemistry.

[B110] Nath D, Rao M (2001). Arificial chaperone mediated refolding of xylanase from an alkalopholic thermophlic *Bacillus* sp. Eur J Biochem.

[B111] Nomura Y, Ikeda M, Yamaguchi N, Aoyama Y, Akiyoshi K (2003). Protein refolding assisted by self-assembled nanogels as novel artificial molecular chaperone. FEBS Lett.

[B112] Daugherty DL, Rozema D, Hanson PE, Gellman SH (1998). Artificial chaperone-assisted refolding of citrate synthase. J Biol Chem.

[B113] Machida S, Ogawa S, Xiaohua S, Takaha T, Fujii K, Hayashi K (2000). Cycloamylose as an efficient artificial chaperone for protein refolding. FEBS Lett.

[B114] Mannen T, Yamaguchi S, Honda J, Sugimoto S, Nagamune T (2001). Expanded-bed protein refolding using a solid-phase artificial chaperone. J Biosci Bioeng.

[B115] Simon SM, Peskin CS, Oster GF (1992). What drives the translocations of proteins. Proc Natl Acad Sci USA.

[B116] Yoshii H, Furuta T, Yasunishi A, Kojima T (1994). Rapid renaturation of denatured and aggregated proteins using liquid paraffin as a pseudolipid bilayer membrane. Biotechnol Bioeng.

[B117] Shinde U, Inouye M (1993). Intramolecular chaperones and protein folding. Trends Biochem Sci.

[B118] Zhu X, Ohta Y, Jordan F, Inouye M (1989). Pro-sequence of subtilisin can guide the refolding of denatured subtilisin in an intermolecular process. Nature.

[B119] Beer H-D, Wohlfahrt G, Schmid RD, McCarthy JEG (1996). The folding and activity of the extracellular lipase of *Rhizopus oryzae *are modulated by a prosequence. Biochem J.

[B120] Tang B, Nirasawa S, Kitaoka M, Marie-Claire C, Hayashi K (2003). General function of N-terminal propeptide on assisting protein folding and inhibiting catalytic activity based on observations with a chimeric thermolysin-like protease. Biochem Biophys Res Commun.

[B121] Rattenholl A, Lilie H, Grossmann A, Stern A, Schwarz E, Rudolph R (2001). The pro-sequence facilitates folding of human nerve growth factor from *Escherichia coli *inclusion bodies. Eur J Biochem.

[B122] Jin H-J, Dunn MA, Borthakur D, Kim YS (2004). Refolding and purification of unprocessed porcine myostatin expressed in *Escherichia coli*. Protein Expr Purif.

[B123] Hahm MS, Chung BH (2001). Refolding and purification of yeast carboxypeptidase Y expressed as inclusion bodies in *Escherichia coli*. Protein Expr Purif.

[B124] Ermolenko DN, Zherdev AV, Dzantiev BB, Popov VO (2002). Antiperoxidase antibodies enhance refolding of horseradish peroxidase. Biochem Biophys Res Commun.

[B125] Xu Q, Xie Z, Ding J, Lin S-X, Xu G (2004). Monoclonal antibodies assisting refolding of firefly luciferase. Protein Sci.

[B126] Ahmed AK, Schaffer SW, Wetlaufer DB (1975). Nonenzymic reactivation of reduced bovine pancreatic ribonuclease by air oxidation and by glutathione oxidoreduction buffers. J Biol Chem.

[B127] Menzella HG, Gramajo HC, Ceccarelli EA (2002). High recovery of prochymosin from inclusion bodies using controlled air oxidation. Protein Express Purif.

[B128] Rudolph R, Lilie H (1996). In vitro folding of inclusion body proteins. FASEB J.

[B129] Wetlaufer DB, Branca PA, Chen G-X (1987). The oxidative folding of proteins by disulfide plus thiol does not correlate with redox potential. Protein Eng.

[B130] Misawa S, Kumagai I (1999). Refolding of therapeutic proteins produced in *Escherichia coli *as inclusion bodies. Biopolymers.

[B131] DeCollo TV, Lees WJ (2001). Effects of aromatic thiols on thiol-disulfide interchange reactions that occur during protein folding. J Org Chem.

[B132] Gough JD, Williams RH, Donofrio AE, Lees WJ (2002). Folding disulfide-containing proteins faster with an aromatic thiol. J Am Chem Soc.

[B133] Shimizu H, Fujimoto K, Kawaguchi H (1999). Refolding of protein using thiol-carrying latex particles. Colloid Surface A.

[B134] Shimizu H, Fujimoto K, Kawaguchi H (2000). Renaturation of reduced ribonuclease A with a microsphere-induced refolding system. Biotechnol Prog.

[B135] Kuhelj R, Dolinar M, Pungercar J, Turk V (1995). The preparation of cataytically active cathepsin B from its precursor expressed in *Escherichia coli *in the form of inclusion bodies. Eur J Biochem.

[B136] Mukhopadhyay A (2000). Reversible protection of disulfide bonds followed by oxidative folding render recombinant hCGβ highly immunogenic. Vaccine.

[B137] Rudolph R, Tschesche H (1990). Renaturation of recombinant, disulfide-bonded proteins from "inclusion bodies". In Modern methods in protein and nucleic acid analysis.

[B138] Gilbert HF (1997). Protein disulfide isomerase and assisted protein folding. J Biol Chem.

[B139] Lilie H, McLaughlin S, Freedman R, Buchner J (1994). Influence of protein disulfide isomerase (PDI) on antibody folding *in vitro*. J Biol Chem.

[B140] Shin H-C, Scheraga HA (2000). Catalysis of the oxidative folding of bovine pancreatic ribonuclease A by protein disulfide isomerase. J Mol Biol.

[B141] Singh RR, Rao AGA (2002). Reductive unfolding and oxidative refolding of a Bowman-Birk inhibitor from horsegram seeds (*Dolichos biflorus*): Evidence for 'hyperreactive' disulfide bonds and rate-limiting nature of disulfide isomerization in folding. Biochim Biophys Acta.

[B142] Hawkins HC, Freedman RB (1995). The effect of denaturants on PDI conformation and activity. Biochem Soc Trans.

[B143] Cabrele C, Fiori S, Pegoraro S, Moroder L (2002). Redox-active cyclic bis(cysteinyl)peptides as catalysts for in vitro oxidative protein folding. Chem Biol.

[B144] Woycechowsky KJ, Wittrup KD, Raines RT (1999). A small-molecule catalyst of protein folding *in vitro* and *in vivo*. Chem Biol.

[B145] Woycechowsky KJ, Hook BA, Raines RT (2003). Catalysis of protein folding by an immobilized small-molecule dithiol. Biotechnol Prog.

[B146] Winter J, Lilie H, Rudolph R (2002). Recombinant expression and in vitro folding of proinsulin are stimulated by the synthetic dithiol Vectrase-P. FEMS Microbiol Lett.

[B147] Hart RA, Lester PM, Reifsnyder DH, Ogez JR, Builder SE (1994). Large scale, *in situ* isolation of periplasmic IGF-I from *E. coli*. Bio/Technology.

[B148] Falconer RJ, O'Neill BK, Middelberg APJ (1999). Chemical treatment of *Escherichia coli*: 3. Selective extraction of a recombinant protein from cytoplasmic inclusion bodies in intact cells. Biotechnol Bioeng.

[B149] Choe W-S, Clemmitt RH, Chase HA, Middelberg APJ (2003). Coupling of chemical extraction and expanded-bed adsorption for simplified inclusion-body processing: Optimization using surface plasmon resonance. Biotechnol Bioeng.

[B150] Choe W, Clemmitt R, Rito-Palomares M, Chase HA, Middelberg APJ (2002). Bioprocess intensification: A radical new process for recovering inclusion body protein. Trans IChemE.

[B151] Choe W-S, Clemmitt RH, Chase HA, Middelberg APJ (2002). Comparison of histidine-tag capture chemistries for purification following chemical extraction. J Chromatogr A.

[B152] Heeboll-Nielsen A, Choe W-S, Middelberg APJ, Thomas ORT (2003). Efficient inclusion body processing using chemical extraction and high gradient magnetic fishing. Biotechnol Prog.

[B153] Lee CT, Morreale G, Middelberg APJ (2004). Combined in-fermenter extraction and cross-flow microfiltration for improved inclusion body processing. Biotechnol Bioeng.

[B154] Ahn JH, Lee YP, Rhee JS (1997). Investigation of refolding condition for *Pseudomonas fluorescens *lipase by response surface methodology. J Biotechnol.

[B155] Lu H, Zhang H, Wang Q, Yuan H, He W, Zhao Z, Li Y (2001). Purification, refolding of hybrid hIFNγ-kringle 5 expressed in *Escherichia coli*. Curr Microbiol.

[B156] Ho JGS, Middelberg APJ (2004). Estimating the potential refolding yield of recombinant proteins expressed as inclusion boidies. Biotechnol Bioeng.

[B157] Trésaugues L, Collinet B, Minard P, Henckes G, Aufrère R, Blondeau K, Liger D, Zhou C-Z, Janin J, van Tilbeurgh H, Quevillon-Cheruel S (2004). Refolding strategies from inclusion bodies in a structural genomics project. J Struct Funct Genomics.

